# Can registry-based auxiliary variables improve handling of missing outcome data in mental health trials? Evidence from a registry-linked randomized study

**DOI:** 10.1186/s13063-025-09346-z

**Published:** 2025-12-09

**Authors:** Amanda Iselin Olesen Andersen, Marit Knapstad, Otto Robert Frans Smith

**Affiliations:** 1 Department of Health Promotion, Norwegian Institute of Public Health, Bergen, Norway; 2 Centre for Evaluation of Public Health Measures, Norwegian Institute of Public Health, Bergen, Norway; 3 Department of Teacher Education, NLA University College, Bergen, Norway

**Keywords:** Missing data, Multiple imputation, Registry data, Randomized controlled trial, Primary care, Depression, Anxiety

## Abstract

**Background:**

Missing outcome data are a pervasive challenge in randomized controlled trials (RCTs) of mental health interventions, potentially biasing effect estimates. Linking survey data with administrative registries offers new opportunities to include auxiliary variables in multiple imputation (MI), but evidence of their added value remains scarce.

**Methods:**

We used data from the Norwegian RCT of Prompt Mental Health Care (PMHC; *N* = 681) which assessed self-report outcomes of depression (PHQ-9) and anxiety (GAD-7) at 6 and 12 months. Registry linkage provided information on prescriptions, consultations, sick leave, and benefits (*N* = 651). We examined whether registry variables were associated with missingness and assessed their correlations with depression and anxiety symptom levels. Further, we compared estimated treatment effects through a linear regression model with treatment group as a predictor across three strategies: (1) complete case analysis, (2) MI with trial data only, and (3) MI with all registry variables in addition to trial data.

**Results:**

Among registry indicators, baseline financial assistance was strongly associated with nonresponse at 12 months. Concurrent registry markers of mental-health consultations and prescriptions were modestly correlated with higher depression and anxiety symptoms at both 6 months and 12 months, whereas prescription use showed modest associations mainly with depressive symptoms (particularly at 12 months) and weaker or null associations with anxiety. The remaining registry variables were weakly associated with missingness and symptom levels, and none were substantially associated with both. Estimated treatment effects were consistent across missing data strategies. Adding registry auxiliaries in MI did not materially change standardized treatment effects of PMHC on depression or anxiety at either time point.

**Conclusions:**

Registry variables showed theoretical and some empirical relevance, but most associations with outcomes and missingness were weak. Including them in MI models did not materially change treatment effect estimates, reinforcing the robustness of the trial’s conclusions. These findings suggest that registry linkages may not always add value, highlighting the importance of prioritizing complete follow-up while identifying contexts where such data could be more impactful.

**Trial registration:**

ClinicalTrials.gov NCT03238872. Registered on August 3, 2017 (retrospectively registered).

**Supplementary Information:**

The online version contains supplementary material available at 10.1186/s13063-025-09346-z.

## Background

Missing outcome data are a major threat to the validity of randomized trials and longitudinal studies, regardless of research design. This problem is particularly common when outcomes are collected through surveys or questionnaires, where nonresponse and attrition are widespread. When outcomes such as symptom measures are unavailable for a substantial proportion of participants, effect estimates can become biased, and generalizability is compromised if not adequately addressed [1–4]. This risk is especially acute when missingness is related to unmeasured participant characteristics or directly to the unobserved outcomes themselves [5–7].

One way to reduce bias under a missing-at-random (MAR) assumption is to incorporate auxiliary variables; that is, variables included in the imputation model (but not in the final analysis) which help capture relationships between observed data, missingness, and the incomplete outcomes [5, 8, 9]. Including variables that predict missingness can improve the plausibility of the MAR assumption, but their impact on estimates depends on whether they are also associated with the incomplete outcomes. Variables that predict missingness but are uncorrelated with the outcomes add little, because they do not inform what the missing values would have been, and in some settings may even exacerbate bias [10–12]. By contrast, variables related to both missingness and the outcomes can substantially reduce bias and increase precision [4, 5, 13]. Simulation studies indicate that associations weaker than about *r* = 0.30–0.40 have limited influence on effect estimates, although weaker predictors may still contribute incrementally when included alongside stronger ones [13, 14]. Accordingly, an inclusive strategy is often recommended, since auxiliary variables are generally unlikely to be detrimental to imputation models and may increase robustness [8, 15, 16]. This is particularly true when auxiliaries are well measured and the outcome is the main incomplete variable [17], though exceptions exist, for example, when auxiliary variables act as colliders [10, 11]. Moreover, even in situations where missingness depends partly on the unobserved values themselves (MNAR), incorporating strong predictors of the outcomes can help to attenuate bias by capturing systematic differences between responders and non-responders [8]. Selection of auxiliary variables should therefore be purposeful and informed by their relevance to both the outcome and the mechanisms leading to missingness.

In longitudinal studies, it is often difficult to identify strong auxiliary variables beyond prior measures of the outcome, since participants are typically completely missing at a given follow-up. Administrative registry data represent a potentially powerful source in this context. These data are routinely collected for entire populations, less prone to recall bias, and available regardless of survey participation [18–20]. Importantly, they may also enrich outcome measurement by capturing information relevant to the outcomes at hand. For example, registries often provide indicators such as healthcare use, prescriptions, and welfare benefits, which may offer indirect insights into mental health status and functioning relevant for studies of mental health.

So far, very few studies have examined the potential of administrative registry data for handling missing outcome data. Population-based surveys have linked registry data to assess selection bias, showing that compared with respondents, non-respondents tend to have poorer health, higher subsequent mortality, and are more likely to have a lower socioeconomic position or precarious employment [21–23]. Nordic registry-linked studies similarly report higher rates of disability pension among non-respondents, often involving mental or musculoskeletal diagnoses [24, 25], while long-term sick leave does not necessarily differ consistently by response status [25, 26]. Some cohort and registry-based studies have explored their use in imputation models, with modest gains in representativeness or precision [27, 28]. Although registry-based RCTs increasingly use registry data for participant identification and clinical endpoints [29, 30], to our knowledge, no randomized controlled trial has tested whether registry-based variables can improve the imputation of self-reported mental health outcomes and reduce potential bias in effect estimates.

In this article, we examine whether registry-based variables can serve as effective auxiliary variables in handling missing outcome data from a randomized controlled trial of Prompt Mental Health Care (PMHC), a Norwegian early intervention program for depression and anxiety. Specifically, we assess the extent to which registry-based variables are associated with the missingness of mental health outcomes, their associations with the outcomes themselves, and whether including them in multiple imputation models changes effect estimates.

## Methods

### Trial design and participants

This is a secondary analysis of a published randomized controlled trial evaluating PMHC compared to treatment as usual (TAU) at 6 and 12 months [31, 32]. The trial was reported according to the CONSORT statement and is registered at ClinicalTrials.gov (NCT03238872). No changes to the design were made after trial commencement. A thorough description of the trial design is provided in the primary evaluation publication [31]. In total, 681 adults with mild to moderate symptoms of anxiety and/or depression were randomized (70:30 ratio; PMHC *n* = 463, TAU *n* = 218). Outcomes were assessed at 6 months (63% response in PMHC, 46% in TAU) and 12 months (54% in PMHC, 43% in TAU).

### Self-reported outcomes

The primary outcomes were symptoms of depression and anxiety, assessed using the Patient Health Questionnaire-9 (PHQ-9) and Generalized Anxiety Disorder-7 (GAD-7), respectively. Both instruments were administered via self-report at baseline, 6 months, and 12 months. The PHQ-9 consists of nine items assessing how often participants experienced core symptoms of depression (e.g., “little interest or pleasure in doing things,” “feeling down, depressed, or hopeless”) over the past 2 weeks [33, 34]. Responses are rated on a four-point scale from 0 (“not at all”) to 3 (“nearly every day”), yielding a sum score from 0 to 27. The GAD-7 uses the same format and response scale to assess seven symptoms of anxiety (e.g., “feeling nervous, anxious or on edge,” “not being able to stop or control worrying”), with total scores ranging from 0 to 21 [33, 35]. Both instruments have shown good psychometric properties, with Cronbach’s alpha of 0.80 (PHQ-9) and 0.83 (GAD-7) in the current sample [31].

Demographic variables included self-reported gender (female/male), age (in years), and education level (primary/lower secondary, upper secondary, or higher education).

### Linked register data

Nationally linked administrative register data were obtained for 651 participants (96% of the total sample). These data were retrieved from the Norwegian Prescription Database (NorPD), the Norwegian Control and Payment of Health Reimbursements Database (KUHR), and the FD-Trygd database (based on data from the Norwegian Labour and Welfare Administration, NAV). Data were constructed for the three time windows aligned with the survey assessments, around baseline, 6 months, and 12 months, enabling time-specific and future auxiliary variables to be included in imputation models.

The register data included:

- Sick leave and social benefits (FD-Trygd): number of sick leave days, receipt of sickness benefits, work assessment allowance, disability pension, and financial assistance (means-tested social welfare support).
- Medication use (NorPD): dispensed prescriptions for antidepressants, anxiolytics, and sedatives within 90 days before or after each follow-up point.
- Healthcare utilization (KUHR): general consultations and mental health–related consultations within 30 days of each survey assessment.

These indicators were chosen to reflect underlying health status, functional impairment, and socioeconomic context, factors that are theoretically linked to both mental health outcomes and the likelihood of missing data. For example, the receipt of work assessment allowance and disability pension are indicators of functional impairment, shown to correlate with depressive symptoms and lower survey participation, particularly when related to mental disorders [24, 25]. Prescription data is also suggested to serve as a proxy for mental health diagnoses and treatment intensity, particularly in populations with known depression or anxiety diagnoses [36]. In addition, consultation data from healthcare registers capture patterns of healthcare utilization, including mental health–related services, which may reflect symptom severity. Thus, all register-based variables were judged conceptually relevant for use as auxiliary variables in imputation.

Although some register indicators (e.g., 12-month data) were temporally located after some of the outcomes of interest (e.g., at 6 months), they were still considered as potential auxiliary variables in imputation models. Including such future data in imputation models is supported in the context of longitudinal studies, where strongly correlated variables can help improve prediction accuracy and reduce bias [37–39].

### Variables associated with missingness and outcome

Separate unadjusted analyses were conducted for each predictor. Specifically, we ran individual logistic regressions for missing outcome data at 6 and 12 months, with odds ratios and p-values used to evaluate strength and significance. In addition, we estimated Pearson correlation coefficients between registry-based variables and PHQ-9 and GAD-7 scores at 6 and 12 months, using pairwise complete cases. Because these analyses were exploratory and aimed at describing patterns rather than testing specific hypotheses, no corrections for multiple comparisons were applied. Together, these analyses provided an overview of the empirical relevance of the registry-based variables.

### Handling of missing data and multiple imputation models

We compared three analytic strategies for handling missing outcome data: (1) complete case analysis (listwise deletion); (2) multiple imputation (MI) with trial data only, including self-reported outcomes (PHQ-9, GAD-7), treatment group, and demographics (gender, age, and education); and (3) MI including both trial data and all available register-based auxiliary variables.

We applied a liberal selection strategy for auxiliary variables, retaining all register-based variables judged conceptually relevant, regardless of statistical significance or effect size. While some authors recommend including only variables with strong associations with either the missingness indicator or the incomplete outcome variable (typically *r* ≥ 0.40–0.50) to optimize efficiency and reduce noise [8, 13, 40], others have shown that even weaker associations may improve imputation accuracy, especially in the presence of high levels of missingness [8, 14–16]. A liberal approach is consistent with recommendations to increase the plausibility of the MAR assumption by incorporating diverse sources of information [8] and with findings that inclusive approaches tend to perform better in improving missing data procedures in realistic settings [15]. To reduce the risk of overfitting and model instability, the number of auxiliary variables remained within the recommended limit of fewer than one-third of complete cases [41].

MI was performed using Multiple Imputation by Chained Equations (MICE), implemented in the *mice* package (version 3.17.0) in R [39]. MICE is a flexible approach that supports variable-specific imputation models, allows the inclusion of auxiliary variables not used in the analysis model, and generates complete datasets suitable for downstream analyses. MI was run separately for each outcome (PHQ-9 at 6 and 12 months, and GAD-7 at 6 and 12 months), resulting in four imputation models. For each model, 100 imputed datasets were generated. Predictive Mean Matching (PMM) was used for continuous variables to preserve realistic distributions and prevent implausible values [42]. The plausibility of imputed values was confirmed by density plots showing broadly similar distributions of observed and imputed data for all outcomes and time points.

### Estimating treatment effects

Treatment effects on PHQ-9 and GAD-7 scores at 6- and 12-month follow-up were estimated using linear regression models run separately for each outcome and time point, with treatment group (PMHC versus TAU) as a predictor. Models were estimated within each of the 100 imputed datasets and pooled using Rubin’s rules [43] to obtain combined effect estimates and confidence intervals. Results across MI strategies were compared to evaluate whether including register-based auxiliary variables affected the magnitude or precision of estimated treatment effects. Standardized treatment effects were calculated by dividing the treatment coefficient by the baseline standard deviation of the corresponding outcome variable, to allow comparability across strategies and outcomes.

All analyses were conducted using R version 4.4.0 [44] and RStudio version 2024.12.1 [45].

## Results

### Variables associated with missingness

In unadjusted logistic regression models (Table 1), several trial- and registry-based variables showed associations with missing outcome data at 6 and 12 months. Among the trial variables, higher baseline PHQ-9 scores were modestly associated with increased odds of missingness at both follow-up points (OR = 1.07, *p* < 0.001; OR = 1.05, *p* = 0.005). Baseline GAD-7 scores showed a somewhat similar pattern but were not statistically significantly associated with attrition. Higher age and education level were protective factors, with education showing a moderate association, especially at 12 months (OR = 0.63, *p* < 0.001). Female gender was weakly protective at 6 months only (OR = 0.70, *p* = 0.033).

**Table 1:** Odds ratios for missing outcomes by time point (*N* = 651)

**Variable**	**Time point**	Missing at 6 months		Missing at 12 months	
		**OR**	***p***	**OR**	***p***
*Trial variables*					
Age	Baseline	0.976	< 0.001**	0.972	< 0.001***
Education level	Baseline	0.723	0.008**	0.634	< 0.001***
Gender	Baseline	0.700	0.033*	0.870	0.405
GAD-7 score	Baseline	1.033	0.094	1.033	0.088
	6 months	-	-	1.039	0.159
	12 months	1.126	0.001**	-	-
PHQ-9 score	Baseline	1.067	< 0.001***	1.052	0.005**
	6 months	-	-	1.026	0.213
	12 months	1.096	0.001**	-	-
*Registry variables*					
Any consultation	Baseline	1.025	0.166	1.010	0.556
	6 months	1.021	0.202	1.017	0.307
	12 months	1.027	0.129	1.024	0.195
Any prescription	Baseline	0.999	0.976	0.999	0.970
	6 months	1.026	0.574	0.982	0.697
	12 months	0.959	0.341	0.931	0.102
Disability pension	Baseline	0.540	0.209	0.832	0.678
	6 months	0.540	0.209	0.832	0.678
	12 months	0.555	0.197	0.647	0.302
Mental health consultation	Baseline	1.070	0.081	1.086	0.041*
	6 months	1.041	0.261	1.069	0.080
	12 months	1.074	0.069	1.018	0.625
On sick leave	Baseline	1.051	0.769	1.129	0.473
	6 months	0.768	0.228	1.085	0.702
	12 months	1.603	0.101	1.123	0.688
Sick leave days	Baseline	1.001	0.692	1.000	0.924
	6 months	0.999	0.452	1.001	0.596
	12 months	1.002	0.328	1.001	0.642
Financial assistance	Baseline	1.384	0.577	10.430	0.025*
	6 months	2.527	0.100	3.454	0.059
	12 months	1.733	0.416	3.268	0.142
Work assessment allowance	Baseline	0.669	0.204	1.000	0.999
	6 months	0.827	0.463	1.067	0.796
	12 months	0.877	0.548	1.039	0.857

All odds ratios are derived from separate unadjusted logistic regression models, each including one predictor at a time

*p < 0.05, **p < 0.01, ***p < 0.001

For registry-based variables, most indicators showed weak or inconsistent associations. For example, receipt of financial assistance around baseline was strongly associated with increased missingness at 12 months (e.g., OR = 10.43, *p* = 0.025), but not at 6 months (OR = 1.38, *p* = 0.577). Similarly, mental health consultations around baseline were statistically significantly associated with missingness at 12 months (OR = 1.09, *p* = 0.041), but not at 6 months. Other registry-based indicators, including prescriptions and sick leave, did not show meaningful associations with attrition.

Group-level description for registry-based variables is provided in the Appendix (Table A1), and indicates very similar distributions across PMHC and TAU, with only small and inconsistent differences observed.

### Variables associated with depression and anxiety outcomes

Correlational analyses (see Tables 2 and 3) identified several variables that were moderately to strongly associated with depression (PHQ-9) and anxiety (GAD-7) symptoms at both 6 and 12 months. Among the trial variables, baseline symptom scores (PHQ-9 and GAD-7) were moderately associated with later symptoms (e.g., *r* = 0.32 for PHQ-9 at 6 months;* r* = 0.26 for PHQ-9 at 12 months; both* p* < 0.001). The strongest correlations, however, were observed for concurrent symptom scores (e.g., PHQ-9 at 6 months with GAD-7 at 6 months: *r *= 0.73, *p* < 0.001; PHQ-9 at 12 months with GAD-7 at 12 months: r = 0.77, *p* < 0.001). In addition, higher baseline education level and female gender were weakly associated with lower symptom scores at 6 and 12 months for PHQ-9, while only education level was statistically significant at 12 months for GAD-7.

**Table 2:** Variables associated with symptoms of depression (*N* = 651)

		PHQ-9 at 6 months		PHQ-9 at 12 months	
**Variable**	**Time point**	***r***	***p***	***r***	***p***
*Trial variables*					
Age	Baseline	0.044	0.396	−0.036	0.526
Education level	Baseline	−0.135	0.009**	−0.168	0.003**
Gender	Baseline	−0.104	0.044*	−0.168	0.003**
GAD-7 score	Baseline	0.118	0.022*	0.087	0.124
	6 months	0.725	< 0.001***	0.509	< 0.001***
	12 months	0.526	< 0.001***	0.772	< 0.001***
PHQ-9 score	Baseline	0.317	< 0.001***	0.261	< 0.001***
	6 months	-	-	0.677	< 0.001***
	12 months	0.677	< 0.001***	-	-
*Registry-variables*					
Any consultation	Baseline	0.003	0.953	−0.026	0.653
	6 months	0.164	0.001**	0.078	0.169
	12 months	0.145	0.005**	0.202	< 0.001***
Any prescription	Baseline	0.055	0.291	0.016	0.783
	6 months	0.152	0.003**	0.139	0.014*
	12 months	0.232	< 0.001***	0.224	< 0.001***
Disability pension	Baseline	−0.053	0.305	0.030	0.600
	6 months	−0.053	0.305	0.030	0.600
	12 months	−0.059	0.253	0.024	0.670
Mental health consultation	Baseline	0.097	0.059	−0.049	0.384
	6 months	0.319	< 0.001***	0.122	0.031*
	12 months	0.282	< 0.001***	0.434	< 0.001***
On sick leave	Baseline	0.007	0.893	−0.100	0.078
	6 months	0.184	< 0.001***	0.054	0.343
	12 months	0.029	0.573	0.176	0.002**
Sick leave days	Baseline	0.092	0.075	−0.039	0.497
	6 months	0.187	< 0.001***	0.063	0.264
	12 months	0.056	0.282	0.142	0.012*
Financial assistance	Baseline	0.074	0.150	0.202	< 0.001***
	6 months	0.145	0.005**	0.248	< 0.001***
	12 months	0.029	0.574	0.057	0.312
Work assessment allowance	Baseline	0.098	0.058	−0.009	0.876
	6 months	0.153	0.003**	0.078	0.167
	12 months	0.224	< 0.001***	0.161	0.004**

Correlations represent separate unadjusted analyses between each predictor and the outcome

*p < 0.05, **p < 0.01, ***p < 0.001

**Table 3:** Variables associated with symptoms of anxiety (*N* = 651)

		GAD-7 at 6 months		GAD-7 at 12 months	
**Variable**	**Time point**	***r***	***p***	***r***	***p***
*Trial variables*					
Age	Baseline	−0.070	0.176	−0.089	0.120
Education level	Baseline	−0.063	0.224	−0.128	0.024*
Gender	Baseline	−0.055	0.287	−0.111	0.052
GAD-7 score	Baseline	0.357	< 0.001***	0.261	< 0.001***
	6 months	-	-	0.583	< 0.001***
	12 months	0.583	< 0.001***	-	-
PHQ-9 score	Baseline	0.178	< 0.001***	0.117	0.039*
	6 months	0.725	< 0.001***	0.526	< 0.001***
	12 months	0.509	< 0.001***	0.772	< 0.001***
*Registry-variables*					
Any consultation	Baseline	−0.006	0.906	−0.068	0.235
	6 months	0.147	0.004**	0.098	0.085
	12 months	0.082	0.111	0.097	0.089
Any prescription	Baseline	−0.032	0.530	−0.090	0.112
	6 months	0.046	0.374	0.033	0.565
	12 months	0.128	0.013*	0.108	0.058
Disability pension	Baseline	−0.019	0.720	−0.031	0.589
	6 months	−0.019	0.720	−0.031	0.589
	12 months	−0.037	0.478	−0.041	0.477
Mental health consultation	Baseline	0.095	0.066	−0.084	0.141
	6 months	0.277	< 0.001***	0.127	0.025*
	12 months	0.257	< 0.001***	0.373	< 0.001***
On sick leave	Baseline	0.033	0.522	−0.113	0.046*
	6 months	0.155	0.003**	0.035	0.541
	12 months	0.019	0.720	0.120	0.035*
Sick leave days	Baseline	0.037	0.474	−0.071	0.214
	6 months	0.193	< 0.001***	0.033	0.566
	12 months	0.076	0.141	0.130	0.022*
Financial assistance	Baseline	0.043	0.410	0.173	0.002**
	6 months	0.053	0.307	0.201	< 0.001***
	12 months	−0.034	0.511	0.095	0.094
Work assessment allowance	Baseline	0.024	0.643	−0.006	0.913
	6 months	0.098	0.058	0.063	0.269
	12 months	0.163	0.002**	0.098	0.085

Correlations represent separate unadjusted analyses between each predictor and the outcome

*p < 0.05, **p < 0.01, ***p < 0.001

Among the registry-based variables, concurrent indicators of mental health consultations and prescription use (at 6 and 12 months) showed the most consistent associations with higher depression and anxiety symptoms. For example, mental health consultation at 12 months was moderately correlated with PHQ-9 symptoms at the same time point (*r* = 0.43, *p* < 0.001) and with GAD-7 (*r* = 0.37, *p* < 0.001). Prescription use around 12 months showed a moderate association with depressive symptoms (*r* = 0.23, *p* < 0.001) but a weaker association with anxiety (*r* = 0.14, *p* = 0.022). Other registry indicators, such as sick leave days, financial assistance, and work assessment allowance, were occasionally associated with symptom severity at some time points, but most of these correlations were weak and inconsistent. In contrast, disability pension and most baseline registry indicators were generally unrelated to symptoms of depression and anxiety.

### Estimated treatment effects across missing data strategies

**Fig. 1:**
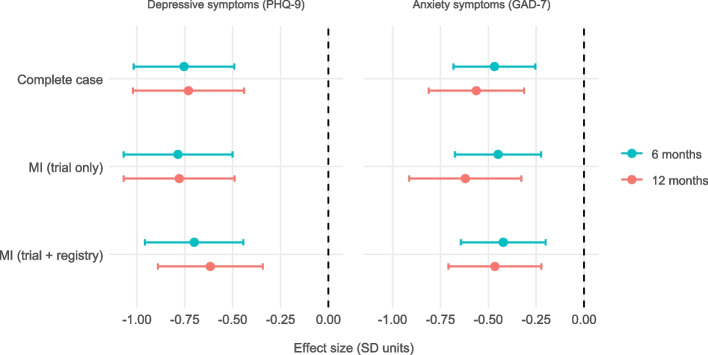
Standardized treatment effects of PMHC on PHQ-9 and GAD-7 at 6 and 12 months, estimated using three different strategies for handling missing outcome data. Points represent estimated effects with 95% confidence intervals. Negative values indicate lower symptom burden in the intervention group. MI = multiple imputations; registry = registry-based auxiliary variables

To examine whether including register-based auxiliary variables improves the handling of missing outcomes data and influences treatment effect estimates, we compared three analytic strategies: (1) complete case analysis, (2) MI using trial data only, and (3) MI including all registry-based auxiliary variables. Table 4 and Fig. 1 present estimated treatment effects of PMHC compared to TAU on PHQ-9 and GAD-7 scores at 6- and 12-month follow-up across the strategies.

**Table 4:** Estimated treatment effects on PHQ-9 and GAD-7 scores using different missing data strategies*

Outcome	Time point	Model	Treatment effect (*b*)	SE	Standardized effect	95% CI
PHQ-9	6 months	Complete case	−3.361	0.597	−0.754	[−1.017, −0.492]
		MI (trial only)	−3.500	0.646	−0.786	[−1.07, −0.501]
		MI (trial + registry)	−3.124	0.586	−0.701	[−0.959, −0.444]
	12 months	Complete case	−3.257	0.660	−0.731	[−1.021, −0.441]
		MI (trial only)	−3.472	0.659	−0.779	[−1.069, −0.49]
		MI (trial + registry)	−2.749	0.623	−0.617	[−0.891, −0.343]
GAD-7	6 months	Complete case	−1.948	0.453	−0.468	[−0.682, −0.255]
		MI (trial only)	−1.869	0.478	−0.449	[−0.675, −0.224]
		MI (trial + registry)	−1.755	0.468	−0.422	[−0.643, −0.201]
	12 months	Complete case	−2.340	0.529	−0.563	[−0.812, −0.313]
		MI (trial only)	−2.578	0.622	−0.620	[−0.913, −0.327]
		MI (trial + registry)	−1.939	0.517	−0.466	[−0.709, −0.223]

*Note that the effect estimates presented here differ from those in our 2020 article [31]. In the present study, we included all participants who met caseness at baseline, defined as PHQ ≥ 10 and/or GAD ≥ 8. By contrast, in the 2020 article we reported effect estimates separately for participants who met caseness for depression (PHQ-9 ≥ 10) and for those who met caseness for anxiety (GAD ≥ 8). Additional, less central, differences are that the current analysis was restricted to participants with valid registry data, excluded the 3-month follow-up, and used a different analytic model (MI + linear regression vs. MLR + piecewise latent growth)

For all outcomes and time points, the estimated effects consistently favored the PMHC group over TAU. Standardized effect sizes ranged from − 0.42 to − 0.79 SD, indicating small to moderate effects in favor of PMHC (i.e., relatively greater reductions in symptoms in the intervention group). Estimates were broadly similar across analytic strategies: complete case and trial-only MI models yielded slightly larger effect estimates, while inclusion of registry-based auxiliary variables produced somewhat more conservative effects (about 0.03–0.16 SD; 6–25% smaller in absolute values, with attenuation a bit more pronounced at 12 months than 6 months). Similarly, standard errors and confidence intervals were largely comparable across analytic strategies, with moderate reductions when registry-based auxiliary variables were included (SE reductions of 2–17% relative to trial-only MI). Overall, the inclusion of registry-based auxiliary variables had a limited impact on the estimated treatment effects or their precision, and the direction and magnitude of effects were robust across all missing-data approaches.

## Discussion

This study examined whether including registry-based auxiliary variables in MI models for missing outcomes would influence treatment effect estimates in a randomized trial of PMHC. The intervention showed benefits for both depression and anxiety outcomes at 6 and 12 months, and the pattern of results was robust regardless of how missing data were handled. Including registry-based auxiliary variables in imputation might support more plausible assumptions about missing data mechanisms and reduce potential bias; in this study, however, adding these variables made only modest changes to the estimated treatment effects compared with complete case and trial-only MI models. These differences were small enough that they are unlikely to alter the practical interpretation of the intervention’s effectiveness, suggesting that the main conclusions remained unchanged. At the same time, the inclusion of registry-based auxiliary variables yielded modest but meaningful gains in precision, with standard errors reduced by up to 17% for some outcomes. Such improvements are non-trivial from an experimental precision perspective. The findings are also valuable in showing when additional data sources do not materially alter conclusions, potentially because missing data may have introduced minimal bias in this particular trial. Taken together, these results may help to refine methodological recommendations, avoid unnecessary model complexity, and direct future efforts towards contexts where such data are more likely to add value.

Our approach aligns with general recommendations to include relevant auxiliary variables in MI to improve the plausibility of the MAR assumption [5, 8, 9]. Prior work has shown that incorporating external data can improve outcome estimation [27, 46] or inform differences between participants and non-participants [21]. However, the added value of auxiliary variables might be context-dependent and rely on their predictive strength and complementarity with existing data. A UK cohort study similarly found that administrative data added little value to imputation models [28]. Notably, while their imputation model already included rich and highly predictive survey-based variables, the available factors in our study, both demographic and register-based, were only modestly associated with both symptom outcomes and missingness.

Several factors may help explain the limited utility of registry-based auxiliary variables in our analyses. Most indicators, such as medication use, sick leave, and healthcare consultations, showed only weak associations with both symptom severity and follow-up attrition. One likely reason is that registry data and self-reported symptoms capture different aspects of mental health: administrative indicators primarily reflect service use or eligibility for benefits, whereas symptom scales assess current subjective distress. Individual variability in help-seeking behavior, the timing of service contact, and day-to-day symptom fluctuations may further reduce alignment between the two. Additionally, several registry variables were relevant only for a minority of participants, reducing their statistical contribution. The trial’s eligibility criteria, reflecting the intervention’s target group, may have constrained the explanatory potential of registry indicators. Because the PMHC trial included only individuals with mild to moderate symptoms, excluding more severe or complex cases, the range of both outcomes and auxiliary variables was restricted. This restriction likely reduced statistical variability at the group level, attenuating the observed associations with symptom levels and dropout risk. Taken together, these considerations highlight that the usefulness of registry-based auxiliary variables depends not only on their availability, but also on their conceptual relevance, population characteristics, and explanatory power in relation to both outcomes and mechanisms of missingness. Viewed this way, these domains also point to practical criteria that may help determine when auxiliary variables are likely to meaningfully strengthen imputation models in other studies.

Registry-based auxiliary variables may be particularly valuable in studies with higher attrition, greater heterogeneity in symptom severity, or, perhaps most important, outcomes more directly aligned with administrative domains (e.g., healthcare use, medication adherence, employment). A promising direction for future work is the systematic collection of short patient-reported outcome measures (PROMs) across primary care services, which could then be made available in registries. Such data would likely correlate more strongly with questionnaire-based outcomes used in trials and thereby enhance the ability to handle missing data and evaluate intervention effects in real-world settings. Evidence from reviews and implementation studies underscores both the potential and the challenges of such integration [47, 48]. Thus, the limited added value observed here should not be taken to imply that registry data are unhelpful in other trials, especially where survey predictors are sparse or attrition is higher.

We adopted an inclusive MI strategy that also allowed time-window variables measured after some outcomes (e.g., 12-month registry indicators when imputing 6-month outcomes) as auxiliary information. While the inclusion of “future” variables in imputation can be debated, using strongly correlated post-outcome auxiliaries can improve imputations without changing the analysis model’s interpretation. Importantly, results were highly consistent across strategies, suggesting this decision did not materially affect conclusions. However, even with extensive auxiliary information, the risk of a missing-not-at-random (MNAR) mechanism cannot be excluded. MNAR may arise either because missingness depends on predictors that were not observed, or because it depends directly on the unobserved outcome values themselves. If non-responders differ from responders in ways not captured by either trial or registry data, some uncertainty will persist in the estimated treatment effects. For instance, participants with high symptom burden might avoid follow-up surveys without showing increased service use, leaving them unobserved in both data sources. Auxiliary variables can help strengthen analytic transparency and increase the plausibility of the MAR assumption, but they cannot eliminate the possibility of bias. The robustness of estimates across strategies in this study should therefore not be taken as evidence that all sources of bias were addressed. Still, missing data mechanisms remain partially untestable. Ultimately, the most effective way to minimize uncertainty in outcome estimation is to ensure high response rates and complete follow-up. Registry data can be a valuable supplement in some studies but cannot replace high-quality outcome data collected directly from participants.

### Strengths and limitations

A key strength of this study is the integration of detailed trial information with highly relevant nationally linked registry data for most of the randomized participants, regardless of survey response status. This enabled a rare and rigorous evaluation of whether administrative data can meaningfully contribute to the handling of missing outcome data in a randomized trial context. The pragmatic design and real-world setting increase the relevance of findings, particularly in mental health research where missing data are common and auxiliary information may be especially valuable. To our knowledge, this is one of the first studies to empirically test this approach in the field of mental health. Furthermore, the analytic strategy was transparent, applying an inclusive approach to auxiliary variable selection that was theoretically grounded and consistent with methodological recommendations. This strengthens the credibility and reproducibility of results while also illustrating the potential and limitations of registry data as auxiliary information in MI.

Nonetheless, several limitations should be noted. All imputations were conducted on the full sample, combining the intervention and control groups. While this ensured sufficient power and stable estimates, it assumes that symptom development and dropout mechanisms operate similarly across groups. However, it is possible that different factors influenced follow-up participation or symptom change in the two conditions in opposite directions. For example, symptom improvement might explain dropout in the intervention group, while ongoing distress or disengagement could drive non-response in the control group. If such group-specific mechanisms exist, pooling the data could obscure important differences and limit the precision of the imputed estimates. Although the treatment group was included in the imputation model, future studies may benefit from stratified imputations. Lastly, generalizability may be limited to settings with similar registry infrastructures, such as the Nordic countries, where linkage is feasible and coverage is high or with different patterns of missingness and follow-up. In other contexts, with less robust administrative systems or different help-seeking patterns, the utility of registry-based auxiliary variables may differ.

## Conclusion

In this PMHC trial, incorporating registry-based auxiliary variables into multiple imputation models had limited impact on treatment effect estimates or their precision. The direction and magnitude of effects were consistent across analytic strategies, supporting the use of both conventional and more inclusive MI approaches in mental health trials. Nonetheless, registry data remains a promising resource for improving the handling of missing data, particularly in studies where such data are more strongly related to missingness or outcomes, or where trial data are less complete or less predictive. However, efforts to maximize response rates at follow-up should remain the primary strategy for addressing missing outcome data.

## Supplementary Information

Supplementary Material 1: Table A1. Descriptives for linked register data.

Supplementary Material 2: Consort.

## Data Availability

The trial data include information linked from Norwegian administrative registries and are subject to legal and ethical restrictions.
