# Supplementary material for: Can registry-based auxiliary variables improve handling of missing outcome data in mental health trials? Evidence from a registry-linked randomized study
Source: Trials. 2025 Dec 9;27:33. doi: 10.1186/s13063-025-09346-z (PMC12802243; doi:10.1186/s13063-025-09346-z)
Supplement: Supplementary file 1 — Supplementary Material 1: Table A1. Descriptives for linked register data. [file 13063_2025_9346_MOESM1_ESM.docx]

# **Appendix**

**Table A1.** Descriptives for linked register data

| **Variable** | **Time point** | **Group** | **n > 0** | **% > 0** | **Mean (overall)** | **Mean (>0)** | **SD (overall)** |
| --- | --- | --- | --- | --- | --- | --- | --- |
| Any consultation ^a^ | Baseline | PMHC | 399 | 89.1 | 4.68 | 5.26 | 4.57 |
|  |  | TAU | 184 | 90.6 | 4.92 | 5.43 | 4.54 |
|  | 6 months | PMHC | 337 | 75.2 | 3.67 | 4.88 | 4.73 |
|  |  | TAU | 143 | 70.4 | 4.10 | 5.82 | 5.41 |
|  | 12 months | PMHC | 309 | 69.0 | 3.38 | 4.89 | 4.35 |
|  |  | TAU | 152 | 74.9 | 4.03 | 5.38 | 4.64 |
| Mental health consultation ^a^ | Baseline | PMHC | 296 | 66.1 | 1.87 | 2.83 | 2.16 |
|  |  | TAU | 153 | 75.4 | 2.10 | 2.78 | 1.91 |
|  | 6 months | PMHC | 164 | 36.6 | 1.00 | 2.72 | 2.16 |
|  |  | TAU | 79 | 38.9 | 1.23 | 3.15 | 2.31 |
|  | 12 months | PMHC | 131 | 29.2 | 0.82 | 2.81 | 2.05 |
|  |  | TAU | 64 | 31.5 | 1.07 | 3.39 | 2.33 |
| Any prescription ^b^ | Baseline | PMHC | 146 | 32.6 | 1.00 | 3.06 | 2.31 |
|  |  | TAU | 77 | 37.9 | 1.01 | 2.68 | 1.89 |
|  | 6 months | PMHC | 143 | 31.9 | 0.78 | 2.45 | 1.81 |
|  |  | TAU | 68 | 33.5 | 0.77 | 2.31 | 1.49 |
|  | 12 months | PMHC | 121 | 27.0 | 0.79 | 2.91 | 1.98 |
|  |  | TAU | 58 | 28.6 | 0.76 | 2.66 | 1.72 |
| Mental health prescription ^b^ | Baseline | PMHC | 101 | 22.5 | 0.45 | 1.99 | 0.97 |
|  |  | TAU | 49 | 24.1 | 0.47 | 1.96 | 0.99 |
|  | 6 months | PMHC | 111 | 24.8 | 0.45 | 1.83 | 0.96 |
|  |  | TAU | 46 | 22.7 | 0.42 | 1.85 | 0.87 |
|  | 12 months | PMHC | 103 | 23.0 | 0.51 | 2.23 | 1.20 |
|  |  | TAU | 40 | 19.7 | 0.42 | 2.15 | 1.06 |
| On sick leave ^c^ | Baseline | PMHC | 144 | 32.1 | — | — | — |
|  |  | TAU | 61 | 30.0 | — | — | — |
|  | 6 months | PMHC | 75 | 16.7 | — | — | — |
|  |  | TAU | 31 | 15.3 | — | — | — |
|  | 12 months | PMHC | 38 | 8.5 | — | — | — |
|  |  | TAU | 15 | 7.4 | — | — | — |
| Sick leave days ^d^ | Baseline | PMHC | 142 | 31.7 | 18.77 | 59.21 | 44.49 |
|  |  | TAU | 61 | 30.0 | 15.57 | 51.82 | 38.44 |
|  | 6 months | PMHC | 75 | 16.7 | 32.04 | 191.41 | 80.44 |
|  |  | TAU | 30 | 14.8 | 23.49 | 158.97 | 64.94 |
|  | 12 months | PMHC | 37 | 8.3 | 8.02 | 97.16 | 39.71 |
|  |  | TAU | 15 | 7.4 | 9.91 | 134.13 | 46.09 |
| Financial assistance ^c^ | Baseline | PMHC | 6 | 1.3 | — | — | — |
|  |  | TAU | 6 | 3.0 | — | — | — |
|  | 6 months | PMHC | 8 | 1.8 | — | — | — |
|  |  | TAU | 6 | 3.0 | — | — | — |
|  | 12 months | PMHC | 5 | 1.1 | — | — | — |
|  |  | TAU | 4 | 2.0 | — | — | — |
| Work assessment allowance ^c^ | Baseline | PMHC | 37 | 8.3 | — | — | — |
|  |  | TAU | 11 | 5.4 | — | — | — |
|  | 6 months | PMHC | 54 | 12.1 | — | — | — |
|  |  | TAU | 17 | 8.4 | — | — | — |
|  | 12 months | PMHC | 77 | 17.2 | — | — | — |
|  |  | TAU | 27 | 13.3 | — | — | — |
| Disability pension ^c^ | Baseline | PMHC | 11 | 2.5 | — | — | — |
|  |  | TAU | 10 | 4.9 | — | — | — |
|  | 6 months | PMHC | 11 | 2.5 | — | — | — |
|  |  | TAU | 10 | 4.9 | — | — | — |
|  | 12 months | PMHC | 14 | 3.1 | — | — | — |
|  |  | TAU | 10 | 4.9 | — | — | — |

Note: PMHC = Prompt Mental Health Care (n = 448); TAU = Treatment As Usual (n = 203).
^a^ counts within ±30 days of each assessment; ^b^ counts within ±90 days of each assessment; ᶜ indicator at the assessment date; ᵈ sick-leave days in the ongoing spell at the assessment date.
